# Implementation of Low Glycemic Index Diet Together with Cornstarch in Post-Gastric Bypass Hypoglycemia: Two Case Reports

**DOI:** 10.3390/nu10060670

**Published:** 2018-05-25

**Authors:** Erminia Lembo, Roberta Lupoli, Paola Ciciola, Annalisa Creanza, Eufemia Silvestri, Gennaro Saldalamacchia, Brunella Capaldo

**Affiliations:** 1 Department of Clinical Medicine and Surgery, Federico II University, 80131 Naples, Italy; erminia.lembo@libero.it (E.L.); paola.ciciola@gmail.com (P.C.); ancreanza@gmail.com (A.C.); miasilvestri@libero.it (E.S.); gsaldala@libero.it (G.S.); 2 Department of Neurosciences, Reproductive and Odontostomatological Sciences, Federico II University, 80131 Naples, Italy; roby.lupoli@gmail.com

**Keywords:** post-bariatric hypoglycemia, cornstarch, gastric bypass, nutrition

## Abstract

Post-bariatric hypoglycemia (PBH) is an increasingly recognized long-term complication of bariatric surgery. The nutritional treatment of PBH includes a high-fiber diet and the restriction of soluble and high-glycemic index carbohydrates; however, these measures are not always enough to prevent hypoglycemia. We evaluated the efficacy of uncooked cornstarch, a low-glycemic index carbohydrate characterized by slow intestinal degradation and absorption, in addition to a high-fiber diet, for the treatment of PBH. We report the cases of two young women suffering from severe postprandial and fasting hypoglycemia following Roux-en-Y gastric bypass (RYGB). The patients underwent Continuous Glucose Monitoring (CGM) before and 12–16 weeks after the administration of uncooked cornstarch (respectively 1.25 g/kg b.w. and 1.8 g/kg b.w.) in addition to a high-fiber diet. In both patients, CGM showed more stable glucose levels throughout monitoring, a remarkable reduction of the time spent in hypoglycemia (<55 mg/dL) both during the day (−11% for both patients) and the night (−22% and −32%), and the improvement of all glycemic variability indexes. Our report, within the limit of only two cases, suggests that the implementation of a dietary intervention through the addition of uncooked cornstarch reduces daily glycemic fluctuations and hypoglycemic episodes in patients with PBH.

## 1. Introduction

Post-bariatric hypoglycemia (PBH) is an increasingly recognized complication of bariatric surgery characterized by low blood glucose levels typically occurring 1–3 h after a meal with the associated autonomic and neuroglycopenic symptoms, which are resolved after glucose ingestion [1].

The real prevalence of PBH is unknown and varies from 1 to ~30% according to definition and methods of diagnosis; i.e., hospitalization data or detection of characteristic symptoms through specific questionnaires [2,3,4,5]. There is also a number of undiagnosed cases due to hypoglycemia unawareness or inadequacy of diagnostic tools [1]. Interestingly, using continuous glucose monitoring (CGM) over five days, Kefurt et al. showed that 75% of patients undergoing Roux-en-Y gastric bypass (RYGB) achieved glucose levels below 3.05 mmol/L [6]. Beside the risk of hypoglycemia, post-bariatric patients present a high glucose variability with post-prandial glycemic peaks followed by glucose nadir [7,8], however, some patients experience hypoglycemia not only in the post-prandial period but also fasting or during the night [6]. In cases where the hypoglycemia does not have a clear post-prandial pattern, other potential causes of hypoglycemia, such as autonomous insulin secretion from an insulinoma, should be investigated [9,10].

The treatment of PBH is based on the increased consumption of dietary fiber to slow carbohydrate (CHO) absorption and restrict soluble and high-glycemic index carbohydrates. However, these dietary measures often yield only modest benefits in patients with severe symptoms, thus requiring the addition of acarbose or, ultimately, drugs inhibiting insulin secretion [11,12]. 

Uncooked cornstarch is a low-glycemic index carbohydrate characterized by a slow intestinal degradation and absorption. For these characteristics, it has been used in the management of conditions associated with a high-risk of hypoglycemia, including glycogen storage diseases, type 1 diabetes, and insulin autoimmune syndrome (Hirata’s disease) [13,14,15]. Thus, cornstarch might be useful to stabilize blood glucose and prevent hypoglycemia also in patients with PBH; however, despite its potential benefits, to date the clinical efficacy of cornstarch in patients with PBH has yet to be assessed. We present two patients suffering from severe hypoglycemia following RYGB in whom the administration of cornstarch, associated with a rich-fiber low-carbohydrate index diet, stabilized glucose profile and reduced hypoglycemic episodes.

## 2. Case Report No. 1

**Figure 1:**
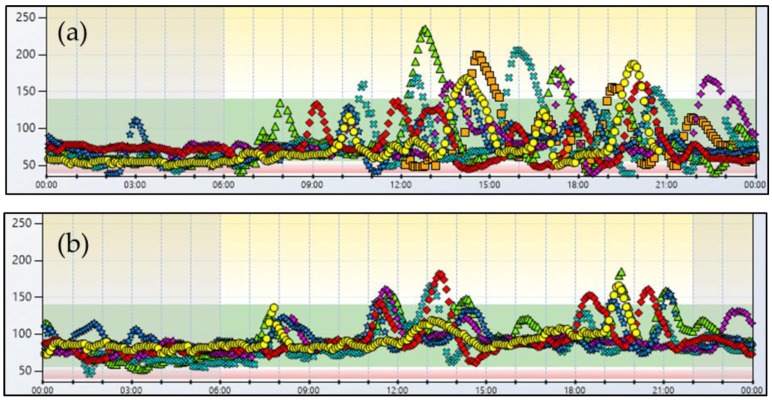
Case 1. 7-day glucose profile before (**a**) and after (**b**) nutritional intervention.

The first subject is a 25-year-old female with a history of obesity who had undergone RYGB 2 year earlier, at which time her BMI was 35.1 kg/m^2^. Two years after surgery, she started to experience frequent episodes of hypoglycemia with tachycardia, sweating, and neuroglycopenic symptoms including confusion, dizziness, and blurred vision. Such symptoms occurred both in the postprandial state and during the night and were so frequent to impact negatively on her working capacity and quality of life. On admission to our Clinic, the patient had lost 35 kg and her body weight was stable. Continuous Glucose Monitoring (CGM, Dexcom G4 PLATINUM) for seven days evidenced ample blood glucose excursions throughout the day, with glucose peaks above 200 mg/dL after meals, followed by a rapid fall below 55 mg/dL (Figure 1a), which were associated with hypoglycemic symptoms as recorded in the patient’s diary. It is interesting to note that low glucose levels occurred also during the night, as demonstrated by the considerable time spent below 55 mg/dL. Based on the continuous glucose profile showing a clear postprandial pattern of hypoglycemia associated with symptoms, we posed the diagnosis of PBH. From CGM, we also calculated the main indexes of glucose variability; i.e., coefficient of variation (CV), standard deviation (SD), and mean amplitude of glycemic excursions (MAGE) that resulted in an increase, as reported in Table 1.

The patient was prescribed a normocaloric (1500 kcal) high-fiber (33 g), low-carbohydrate index (55) diet, which was divided into three meals and three snacks to avoid postprandial blood glucose peaks and prolonged fasting intervals (see Appendix A). Multivitamin and mineral supplements were also prescribed on the basis of biochemical analysis showing vitamin D (19 ng/mL) and iron (ferritin 10 ng/mL) deficiency.

Following nutritional therapy, the patient demonstrated a modest improvement of hypoglycemic symptoms but asthenia and tiredness in the early morning associated with fingerstick glucose level in the low range (<70 mg/dL) persisted. We supplemented the diet with a starting dose (30 g) of uncooked cornstarch administered before going to bed, which was progressively increased to 85 g/die (30 g in the morning and 55 g at bedtime; 1.25 g/kg b.w.) on the basis of the fingerstick home glucose profile. CGM, performed 12 weeks after beginning the cornstarch administration, showed a remarkable reduction in glucose variability indexes and postprandial glycemic peaks. Furthermore, the overall time spent in hypoglycemia (IG <55 mg/dL) decreased significantly. Of particular relevance is the reduction of the time spent in hypoglycemia during the night (from 23 to 1%; from 9.5 h to 35 min) (Figure 1b). No side effect was recorded after cornstarch ingestion except for a slight abdominal distension during the first days of treatment. 

## 3. Case Report No. 2

**Figure 2:**
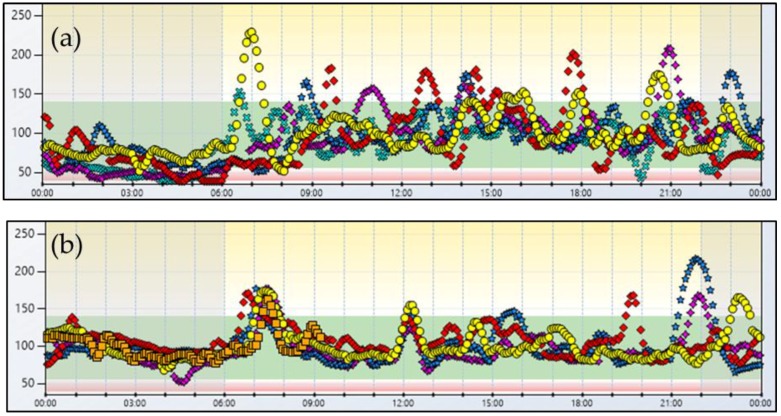
Case 2. 5-day glucose profile before (**a**) and after (**b**) nutritional intervention.

The second patient is a 34-year-old female with a history of obesity since childhood. Over the years, the patient had frequently undergone hypocaloric regimens and/or pharmacologic treatment for obesity, obtaining a consistent weight loss followed inexorably by weight regain. At age 28 years, due to the persistence of severe obesity (BMI 41.2 kg/m^2^) the patient underwent RYGB. One month after surgery, a spontaneous pregnancy was diagnosed. During pregnancy the patient lost ~70 kg, reaching 67 kg at the end of pregnancy. No complication occurred during gestation and a healthy child was born pre-term (36 week) by caesarean section. Five years after RYGB the patient came to our Clinic complaining of tremors, palpitations, and sweating. She also complained of neuroglycopenic symptoms such as profound tiredness, weakness, confusion, disorientation, and blurred vision. These symptoms appeared 1–2 h after meals, especially after carbohydrate-rich meals but also upon awakening. On admission, the patient was in good nutritional conditions, had no sign of vitamin and/or micronutrient deficiency, and a stable body weight (BMI 24.8 kg/m^2^). The CGM documented a pattern of postprandial glycemic peaks followed by hypoglycemia; notably, as for patient 1, it also demonstrated that 33% (11.4 h) of the night was spent at glucose levels below 55 mg/dL (Figure 2a). As described for the previous patient, a high-fiber, low carbohydrate index diet was prescribed in association with uncooked cornstarch (see Appendix A). A starting dose of cornstarch (40 g) was added before going to sleep, which was progressively increased up to 150 g/day (50 g in the morning, 50 g during the afternoon and 50 g at bedtime, equivalent to 1.8 g/kg b.w.). The CGM performed after 24 weeks of nutritional management showed a substantial improvement in her glucose profile with a reduction of the time spent in hypoglycemia (<55 mg/dL) both during the day (from 11% to 0%) and during the night (from 33% to 1%) (Figure 2b). Accordingly, the indexes of glucose variability were remarkably lower than those observed before the nutritional intervention (Table 1). The patient reported feeling much better and that hypoglycemic symptoms had become less severe and much less frequent.

## 4. Discussion

Bariatric surgery (BS) is considered the most effective treatment for obesity since it promotes durable weight loss, improvement/remission of obesity-related comorbidities, reduction of mortality and improvement of quality of life [16,17]. Interestingly, the improvement of the metabolic status occurs very early after surgery, suggesting that weight-independent factors may play a role in achieving metabolic improvement [16,18]. Beside these clinical benefits, BS is reported to have some risks and complications both early after surgery or in the long term. PBH is an emerging long-term complication of BS, particularly of procedures modifying GI anatomy and, consequently, the absorption of nutrients. The growing number of reported cases of PBH is due in part to the increasing number of bariatric procedures performed worldwide, but also due to the introduction in clinical practice of the glucose monitoring systems, which provide moment-to-moment information on glucose levels over the course of days or weeks [5]. Of note, some studies have shown that CGM detected more patients with clinically significant hypoglycemia compared with provocative tests [6,19], underlying the usefulness of this tool in patients with PBH. 

PBH has been most often associated with RYGB but there are some reports that note it can also occur after a sleeve gastrectomy [4,20]. Both our patients had RYGB surgery and started to experience hypoglycemic symptoms two and five years after the intervention, respectively. Their CGM record evidenced the classical pattern of rapid increases in glucose concentration after meals followed by sharp falls below 55 mg/dL. Of particular clinical relevance is the finding that both patients also experienced hypoglycemia during the night, thus explaining their sense of tiredness upon awakening.

The pathophysiology of PBH is still a matter of debate. Several mechanisms have been postulated and include: (1) increased incretin levels, namely GLP-1 and GIP that cause an excessive insulin secretion [21,22]; (2) restoration of the β-cell sensitivity to GLP-1 [23]; (3) persistence of the obesity-driven β-cell hyperfunction despite weight loss [23,24]; (4) increased tissues’ insulin sensitivity consequent to weight loss [1,25]; and, (5) abnormal response of counterregulatory hormones [21,26,27]. With regard to the last mechanism, it is important to remind that GLP-1 has a glucagonostatic effect, which could be potentiated under conditions of recurrently elevated GLP-1 levels. Thus, it is tempting to speculate that the low fasting glucose levels found in our patients could be the result of an inappropriate glucagon-to-insulin ratio that may reduce endogenous glucose production. Another factor possibly contributing to fasting hypoglycemia is a reduction of gluconeogenic substrates. This hypothesis is supported by a study by Lafèrrere et al., who showed that lower levels of plasma aminoacids were present in patients undergoing RYGB compared to diet-treated patients, despite an equivalent decrease in body weight [28].

Several therapeutic options have been proposed for treating PBH [11,12]. To prevent an excessive rise in post-meal blood glucose and, hence, in insulin secretion, the first measure is the adoption of a high fiber, low-glycemic index diet fractioned in frequent small meals. If patients do not respond to nutritional treatment, acarbose is added and, eventually, drugs inhibiting insulin secretion (verapamil, diazoxide or octreotide) which, however, are not devoid of side effects. 

In our patients, we decided to implement nutritional measures by adding uncooked cornstarch, a source of slowly releasable carbohydrates, which produces low glucose peaks and maintains blood glucose stable during fasting. Combining cornstarch with a high-fiber low-glycemic index diet, our patients showed a more stable glucose profile, lower postprandial peaks, and lower hypoglycemic episodes both during the day and the night. All of this resulted in a reduction of glucose variability, a clinically relevant finding given the link between high glucose variability and increased oxidative stress [7]. However, the relative contribution of high-fiber low-glycemic index diet vs. cornstarch in improving overall glucose profile cannot be appreciated with this approach. Indeed, the reduction of nocturnal hypoglycemia is mainly attributable to the bedtime administration of cornstarch, given its ability to maintain normal glucose levels up to 7 h, as shown in patients with glycogen storage diseases [13]. The preliminary positive findings obtained in these two patients encourage us to test more systematically the efficacy of this approach in patients with PBH.

Notably, the clinical improvement was achieved without any relevant side effects. Our patients did not observe any discomfort following cornstarch ingestion, such as abdominal distension or increased flatulence, probably because of the relatively small dose of cornstarch required (lower than that commonly used for the management of glycogen storage diseases). 

## 5. Conclusions

In conclusion, our report, within the limit of only two cases, indicates that adding cornstarch to a high-fiber low-glycemic index diet reduces glycemic fluctuations and hypoglycemic episodes in patients with PBH. This well-tolerated, low-cost intervention should be pursued as a further step in the nutritional management of PBH before considering pharmacologic therapy.

## Figures and Tables

**Table 1:** Time spent at different glucose levels and indexes of glucose variability obtained through Continuous Glucose Monitoring (CGM) before and after nutritional management.

	Case 1			Case 2		
	Before	After	Δ %	Before	After	Δ %
Mean IG ^1^ (mg/dL)	81	92	+13.6	93	101	+8.6
% time > 140 mg/dL	7	4	−3	8	7	−1
% time in range 70–140 mg/dL	49	88	+39	72	92	+20
% time ≥ 55 and <70 mg/dL	32	7	−25	9	1	−8
% time < 55 mg/dL	12	1	−11	11	0	−11
% nightime < 55 mg/dL	23	1	−22	33	1	−32
SD ^2^ (mg/dL)	32	21	−34.4	32	23	−28.1
CV ^3^ (%)	39	23	−16	34	22	−12
MAGE ^4^ (mg/dL)	35.4	23.6	−33.3	35.6	24.6	−30.9

^1^ IG = interstitial glucose, ^2^ SD = standard deviation, ^3^ CV = coefficient of variations, ^4^ MAGE = mean amplitude of glucose excursions.

## Appendix A

### Appendix A.1. High-Fiber Low-Glycemic Index Diet for the Nutritional Management of Patients with Post-Bariatric Hypoglycemia (PBH)

The energy content of the diet was ~1500 kcal/die. The diet composition was as follows: 48% carbohydrate, 33% lipid, 19% protein, fiber 33 g, soluble carbohydrate 40 g (10% total calories). The glycemic index was 55.

### Appendix A.2. An Example Day

- *Breakfast*
  - Coffee or tea or herbal teas or skimmed yogurt 125 g + cornflakes (or oatmeal or wholemeal rusks) 30 g.

- *Lunch*
  - Pasta (or brown rice) 40 g + legumes (lentils, beans, chickpeas) 50 g + parmesan cheese 10 g,
  - Vegetable (spinach, zucchini, beets, green beans, tomato, pumpkin, lettuce) 150 g,
  - Fresh fruit (apple, pear, peach, apricot, orange, kiwi) 150 g.

- *Dinner*
  - Meat 120 g,
  - Vegetables (spinach, zucchini, beets, green beans, tomato, pumpkin, lettuce) 150 g,
  - Whole brain bread 80 g.

- *3 Snacks (mid-day, mid-afternoon, bedtime)*
  - Fresh fruit 150–200 g,
  - Crackers 25 g,
  - 2 toast bread + speck (or fresh cheese or tuna fish) 30 g.

- *Seasoning for the day*
  - Extra-virgin olive oil (30 g).
